# Ten lessons for data sharing with a data commons

**DOI:** 10.1038/s41597-023-02029-x

**Published:** 2023-03-06

**Authors:** Robert L. Grossman

**Affiliations:** University of Chicago, Center for Translational Data Science, Chicago, IL 60615 USA

**Keywords:** Computational platforms and environments, Research data, Data publication and archiving

## Abstract

A data commons is a cloud-based data platform with a governance structure that allows a community to manage, analyze and share its data. Data commons provide a research community with the ability to manage and analyze large datasets using the elastic scalability provided by cloud computing and to share data securely and compliantly, and, in this way, accelerate the pace of research. Over the past decade, a number of data commons have been developed and we discuss some of the lessons learned from this effort.

## What is a Data Commons?

### Cloud computing has made it much easier to store and analyze large datasets, but it is still a challenge to share data effectively with a research community in a way that accelerates research

Since the end of the 16th century, experimental science has been driven by conducting experiments, collecting data, and analyzing it. With the development of low cost sensors, high throughput instruments, and inexpensive storage to hold the data they produce, a new paradigm for scientific discovery has emerged. The *sharing of data*, and its reanalysis in different contexts, with different aims, and in conjunction with other datasets to create new hypotheses and new scientific discoveries is part of what is sometimes called the fourth paradigm of science^1^. (The other three paradigms are: experimental science, theoretical science and simulation science.) In this article, we discuss the role of software platforms called data commons in supporting the fourth paradigm of science and some lessons learned from ten years of experience developing and operating data commons.

As we will describe below, a data commons is a shared resource to support a scientific community. Some of the challenges with shared resources were identified in 1968 when Joseph Hardin published an article in Science called the *The Tragedy of the Commons*^2^ that focused attention on problems arising when a shared finite resource is used by a community. The governance structure is critical. About forty years later in 2009, Elenor Ostrom received the Nobel prize in Economic Sciences^3^ for her work about the governance of the commons^4^.

Following Ostrom, we define a *commons* as a natural, cultural or digital resource accessible to all members of a community, or more broadly of a society. Importantly, a commons is held through a partnership, a not-for-profit, or other entity, for the benefit of a community, but not owned privately for commercial gain^4^.

For the purposes here, we view a *data commons* as a software platform that co-locates: 1) data, 2) cloud-based computing infrastructure, and 3) software applications, tools and services to create a governed resource for managing, analyzing, and sharing data with a community^5^. **Briefly, a data commons is a cloud-based software platform with a governance structure that allows a community to manage, analyze and share its data**. We discuss some of the differences between data commons and data repositories towards the end of the paper.

A related concept is a data mesh (also known as a data ecosystem). A *data mesh* is a collection of data commons, cloud-based computational resources, and other cloud-based resources that interoperate using a common set of core software services and a hybrid governance model.

Examples of data commons and similar platforms include: the NCI Genomic Data Commons (GDC)^6^, the NHLBI BioData Catalyst data platform (https://biodatacatalyst.nhlbi.nih.gov/), the NHGRI Genomic Data Science Analysis, Visualization and Informatics Lab-space (AnVIL)^7^, the NIH Common Fund Data Ecosystem^8^, the All of Us Research Hub^9^, the Australian Research Data Commons^10^, the Elixir Data Platform^11^, the BloodPAC Data Commons^12^, the NIBIB Medical Imaging and Data Resource Center (MIDRC)^13^, the Veterans Precision Oncology Data Commons^14^, and the NIH Kids First Data Resource (https://kidsfirstdrc.org/).

## Why Build Data Commons?

There are several main reasons research projects build data commons.

1. **The functionality is compelling**. Modern cloud computing provides elastic, on-demand, pay-as-you-go computing that can be used to provide compelling functionality in a data commons and accelerate research over the data in the commons. In particular, although there is a learning curve, cloud computing can manage large data (at the petabyte scale), provide large scale compute, and provide specialized scalable services for querying and analyzing data. More generally, data commons can make important data and its interactive exploration more widely available to the scientific community, including to less technically sophisticated users. The most important reason for building a data commons is that the functionality provided by a data commons is compelling.
2. **To speed the pace of research discoveries**. By having the commons curate and processes the data once for a particular research community, it enables individual researchers and research groups to proceed more quickly to analyzing data to investigate particular hypotheses, since each group doesn’t have to curate the data, process the data with a uniform set of pipelines, and quality check the results. This work can be done once by the commons.
3. **To create network effects**. Another reason to build data commons is be part of a larger data ecosystem or data mesh containing multiple commons, computing platforms, and knowledgebases in order to take advantage of network effects as more commons are built and more users access data from the resources in the mesh. As an example, a commons in a data mesh can participate in federated machine learning with other commons and data resources via its APIs, while adhering to its governance, security and privacy policies.
4. **To host data that is too large to be managed easily by research groups**. As the size of the data grows, it becomes more and more difficult for each research group to develop and operate their own computing infrastructure. A data commons is often used to manage the large experimental data and process it to produce derived datasets that are easier to analyze by individual scientists and research groups.
5. **To reduce cost**. Funds for science are always limited and tough choices must be made. By investing in a centralized commons, the cost for managing, curating and processing data to produce data products for a research community can be done once centrally to reduce the overall costs of a research program or initiative. In practice, with very large datasets, the cost of funding each research group to build their own data platform can be so high that the only practical way to distribute the data to the research community is through a centralized data commons.
  Although, it can be more expensive to store data in the cloud than on premise, as the size of the data increases, as the number of different groups using the data increases, and as the percent of the data needed by research groups increases, having a single copy of the data in the cloud is less expensive for the sponsor than separately funding each research group to manage the data separately. Another consideration affecting costs are any egress charges when transferring data out of public clouds. For large datasets that are downloaded by a large number of users, these can be expensive. For this reason, it can be useful to provide copies of large datasets in private clouds where the egress charges may be much less. This is done, for example, in the AnVIL data platform for selected datasets^7^.
6. **To protect sensitive data**. Sometimes data commons are built because research data is so sensitive that it must be protected within an enclave for it to be safely shared. Examples include the All of Us Research Hub^9^ and the Veterans Precision Oncology Data Commons^14^. For example, the data may be in a commons and not available to researchers directly but only through specific integrated software applications (software as a service) that are designed to protect the confidentiality of the data or through virtual desktop infrastructure which restricts the ability to download or otherwise access the data.

As an example, data commons, such as the NCI Genomic Data Commons^6,15^, have made petabytes of curated, harmonized data availability to a research community and “democratized access to cancer genomics data,” which until the launch of the GDC in 2016 was available only to the largest research organizations that had the resources and expertise to analyze petabyte scale data. With the GDC and its open APIs^16^, researchers could access the GDC’s data products in cloud computing environments or on their own local resources for further analysis or integrative studies.

The emergence and adoption of cloud computing over the past decade^17^ has made developing data commons and data meshes much easier. Today, cloud computing is the key technology that has enabled the current generation of data commons.

### A data gap

Although the large and increasing amount of data being generated in biology, medicine and healthcare is well documented^1,17,18^, the amount of well-curated, harmonized data is often not noted. This creates a “data gap” that impedes research. Data commons are designed in part to close this gap and allow research communities to create curated, harmonized data sets to accelerate their research.

## The Success of the NCI Genomic Data Commons

One of the more popular data commons is the NCI Genomic Data Commons^6,15^. As reported in^6^, the GDC contains over 2.9 PB of curated, harmonized cancer genomics data from over 60 projects (as of February 2021). Each month over 50,000 unique researchers use the system and over 1.5 PB of data are accessed^6^. As a rough measure of its popularity, the membership of the American Association of Cancer Research is about 50,000.

The data from the different projects is curated with respect to a single data model and each month on average over 25,000 bioinformatics pipelines are run over the data to create a harmonized set of data products that are analyzed with a common set of bioinformatics pipelines^15^. In contrast, it is typical to bring together cancer genomics data from different projects that are analyzed by different groups using different pipelines, which can make subsequent integrative analysis much more challenging and problematic. The GDC has a user interface that enables interactive graphical exploration of the data, with the ability to download publication quality graphics.

Perhaps the most important reason for its popularity is that the GDC makes it easy for researchers to access its data and make new research discoveries with much less effort than if they were to analyze the raw data themselves, as it usually required with a traditional data repository.

The GDC lists over 100 high impact publications that have been written using the data products it makes available to the cancer genomics research community.

## Ten Lessons About Data Commons

### Lesson 1. Build a commons for a specific community with a specific set of research challenges

Although there are a few data repositories that serve the general scientific community that have proved successful, in general data commons that target a specific user community have proven to be the most successful. The first lesson is to build a data commons for a specific research community that is struggling to answer specific research challenges with data. As a consequence, a data commons is a partnership between the data scientists developing and supporting the commons and the disciplinary scientists with the research challenges.

### Lesson 2. Successful commons curate and harmonize the data

Successful commons curate and harmonize the data and produce data products of broad interest to the community. It’s time consuming, expensive, and labor intensive to curate and harmonize data, by much of the value of data commons is centralizing this work so that it can be done once instead of many times by each group that needs the data. These days, it is very easy to think of a data commons as a platform containing data, not spend the time curating or harmonizing it, and then be surprised that the data in the commons is not used more widely used and its impact is not as high as expected.

### Lesson 3. It’s ultimately about the data and its value to generate new research discoveries

Despite the importance of a study, few scientists will try to replicate previously published studies. Instead, data is usually accessed if it can lead to a new high impact paper. For this reason, data commons play two different but related roles. First, they preserve data for reproducible science. This is a small fraction of the data access, but plays a critical role in reproducible science. Second, data commons make data available for new high value science.

### Lesson 4. Reduce barriers to access to increase usage

A useful rule of thumb is that every barrier to data access cuts down access by a factor of 10. Common barriers that reduce use of a commons include: registration vs no-registration; open access vs controlled access; click through agreements vs signing of data usage agreements and approval by data access committees; license restrictions on the use of the data vs no license restrictions.

### Lesson 5. Data curation and developing interactive user interfaces is expensive

The largest costs of developing and operating a data commons are: i) the costs of data curation and data harmonization; and, ii) the costs of developing easy to use, interactive front ends for exploring and analyzing the data.

### Lesson 6. Support an ecosystem of applications, not just a single system

The most successful commons have open APIs, enabling the community to build utilities, libraries, and tools make the data usable and accessible.

### Lesson 7. Security and compliance for data commons are expensive

The policies, procedures and controls required for security, compliance, and regulatory support are expensive and time consuming to develop and to operate and, in almost all cases, are underfunded.

### Lesson 8. It’s not easy to predict what archived data will lead to great science

Some datasets in a commons tend to be very frequently downloaded, with others much less frequently downloaded, and with the distribution following a Zipfian or other power law distribution^19^. This might lead one to advocate saving operational costs by only hosting the more popular datasets. On the other hand, over the long term, interesting, and sometimes, quite interesting, new science may result from further use and analysis of the less popular datasets.

### Lesson 9. Over time, the value of data commons will grow if it is part of a data mesh

Data commons, at least as we have defined them here, are designed to support a particular research community. In contrast, a data mesh (also known as a data ecosystem) is a hybrid architecture, consisting of some common services that enable a collection of independently managed and governed data platforms to interoperate.

### Lesson 10. Resist the temptation to build a cloud-based walled garden

It is tempting when building a data platform or data commons to take the attitude that everyone should contribute data to your platform, but there is not a good reason, and only risk, if you enable your data to leave your system. As an alternative, if data access needs to be restricted, consider developing trust relationships with other data platforms and sharing data and interoperating with them.

Data commons differ from *traditional* data repositories in several ways. First data commons have FAIR APIs^20^ for discovering and accessing the data they manage (versus user interfaces for downloading data) and have integrated tools for exploring and analyzing data. Of course, more recent data repositories are increasingly being built with FAIR APIs. Also, data commons curate the data submitted to them and in some cases harmonize it by analyzing it with a common set of analysis tools.

**Fig. 1:**
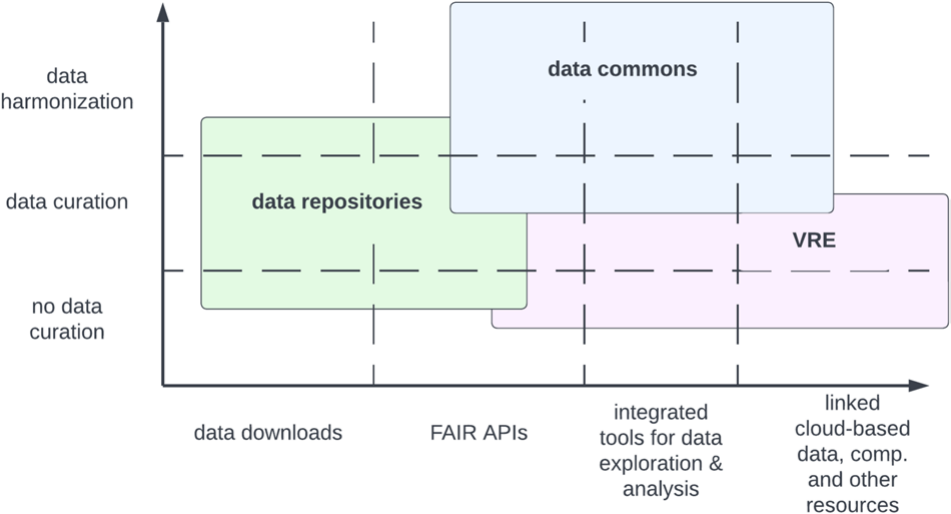
Some of the differences between data repositories, data commons and virtual research environments. Note the overlap.

A related concept is a science gateway or virtual research environment, which following the definition in^21^, we define as a “web-based enterprise information systems that provide scientists with customized and easy access to community-specific data collections, computational tools and collaborative services on e-Infrastructures.” Historically, a science gateway was a web-based portal that brought together data, computational and other resources for a scientific community. These days, science gateways tend to be cloud-based with FAIR APIs. See Fig. 1. As the figure shows, there is not a rigid separation between data repositories, data commons, and virtual research environments, but instead some overlap.

Science and data clouds, such as the European Open Science Cloud^22,23^, tend to integrate a wider variety data and computational resources and serve a much broader range of scientific communities.

## Summary and Conclusion

Large datasets are now prevalent throughout almost all areas of scientific research and cloud computing is increasingly being used to manage and analyze the resulting data. A data commons is a cloud-based computing resource with a governance structure that allows a community to manage, analyze and share its data.

Data commons have emerged in part to address the data gap – the gap between the large amount of data available and the small amount of data that can be easily used to formulate new hypotheses, to make new discoveries, and to build machine learning and AI models.

This point is worth emphasizing. **Despite, the availability of raw generated data and large scale cloud computing infrastructure, science remains** ***data limited*, since the available data needs to be carefully curated and harmonized before it is useful. Commons support this important activity so that research questions can be more efficiently tackled by a research community**.

Looking towards the future, a core set of microservices are emerging^24^ that enable data meshes (data ecosystems) consisting of multiple data commons, cloud-based computing platforms, knowledgebases, and other resources to interoperate. The hope is that making data also available through interoperating with data meshes will further accelerate the pace of research.
